# Serum retinol-binding protein 4 levels in polycystic ovary syndrome

**DOI:** 10.1530/EC-19-0116

**Published:** 2019-05-03

**Authors:** Shilpa Lingaiah, Laure Morin-Papunen, Terhi Piltonen, Inger Sundström-Poromaa, Elisabet Stener-Victorin, Juha S Tapanainen

**Affiliations:** 1 Department of Obstetrics and Gynaecology, PEDEGO Research Unit, Medical Research Centre, University of Oulu and Oulu University Hospital, Oulu, Finland; 2 Department of Women’s and Children’s Health, Uppsala University, Uppsala, Sweden; 3 Department of Physiology and Pharmacology, Karolinska Institutet, Stockholm, Sweden; 4 Department of Obstetrics and Gynaecology, University of Helsinki and Helsinki University Hospital, Helsinki, Finland

**Keywords:** polycystic ovary syndrome (PCOS), retinol-binding protein 4 (RBP4), adipokines

## Abstract

**Objective:**

Serum levels of retinol-binding protein 4 (RBP4), an adipokine thought to affect systemic insulin sensitivity, were compared between women with polycystic ovary syndrome (PCOS) and non-PCOS controls to evaluate the association of RBP4 with clinical, hormonal and metabolic parameters of PCOS.

**Subjects and methods:**

Serum RBP4 levels were analysed in 278 women with PCOS (age range 18–57 years) and 191 non-PCOS controls (age 20–53 years) by enzyme-linked immunosorbent assay.

**Results:**

Serum levels of RBP4 were increased in women with PCOS compared with control women in the whole population (45.1 ± 24.0 (s.d.) vs 33.5 ± 18.3 mg/L, *P* < 0.001). Age-stratified analysis showed that serum RBP4 levels were increased in women with PCOS aged ≤30 years compared with controls (47.7 ± 23.5 vs 27.1 ± 10.4 mg/L, *P* < 0.001), whereas no significant differences were seen in the other age groups. No significant correlations of RBP4 were seen with either steroids or indices of insulin resistance.

**Conclusions:**

Although serum RBP4 levels were increased in younger women with PCOS compared with age-matched non-PCOS controls, RBP4 does not seem to be a good marker of insulin resistance or other metabolic derangements in women with PCOS.

## Introduction

Polycystic ovary syndrome (PCOS) is the most common endocrine disorder in women of reproductive age, with an estimated prevalence of 6–15%, depending on the criteria used for diagnosis (1). It is a heterogeneous disorder with several metabolic and cardiovascular health implications. Women with PCOS commonly suffer from chronic anovulation, infertility, hyperandrogenaemia, obesity, dyslipidaemia and low-grade chronic inflammation (2). Insulin resistance is a common feature of PCOS and women with the syndrome are more insulin resistant than expected for their BMI (3). Furthermore, the presence of central obesity has a detrimental effect on insulin resistance levels.

The aetiology of PCOS is multifactorial and complex. It is postulated that adipose tissue dysfunction plays a significant role in the metabolic abnormalities observed in affected women (4). Though several studies have been carried out to investigate the possible role of various adipocytokines in the pathogenesis of PCOS (5, 6, 7), it is not clear whether they have a direct association with PCOS. Adipose tissue acts as an endocrine organ secreting various adipokines including retinol-binding protein 4 (RBP4), which is mainly synthesised by hepatocytes and adipose tissue. RBP4 is an adipokine with a possible detrimental effect on insulin sensitivity. Indeed, RBP4 is thought to act via alterations in insulin signalling in muscle, inhibiting glucose uptake and interfering with insulin-mediated suppression of glucose production in the liver (8, 9).

Studies have shown that circulating RBP4 levels correlate with the magnitude of insulin resistance in obese subjects and in those with impaired glucose tolerance and type 2 diabetes mellitus (10, 11). However, data concerning the circulating concentrations of RBP4 in women with PCOS are conflicting. Besides unchanged levels of RBP4, increased as well as decreased levels have been reported. Elevated levels of RBP4 have been observed in both lean and overweight/obese women with PCOS (12), while one study revealed higher RBP4 levels only in obese women with PCOS (13). In contrast, lower levels of RBP4 have been reported in lean women with PCOS (14) whereas other investigators have found no difference in the concentrations of RBP4 in women with PCOS compared with BMI-matched controls (15, 16). Results concerning an association between serum RBP4 levels and insulin resistance in PCOS have also been conflicting, as a few investigators have reported no association (14, 15) while others have reported that RBP4 levels correlate positively with insulin resistance, but not PCOS *per se* (17, 18). Up to now there have been no studies in which the levels of RBP4 at different ages during reproductive life in women with PCOS vs non-PCOS women have been compared.

The aim of the present study was to compare the serum levels of RBP4 in women with PCOS vs non-PCOS women at different ages during reproductive life and to evaluate their associations with clinical, hormonal and metabolic parameters.

## Subjects and methods

### Study population

The study population consisted of 278 women with PCOS (age range 18–57 years) and 191 non-PCOS controls (age 20–53 years) who participated in six Nordic PCOS studies: four studies in Finland and two in Sweden (19, 20, 21, 22, 23, 24). PCOS was diagnosed according to the European Society of Human Reproduction and Embryology/American Society for Reproductive Medicine (ESHRE/ASRM) consensus definition (25). Ovarian morphology was assessed by means of transvaginal ultrasonography in all subjects. Biochemical hyperandrogenism was defined as serum testosterone ≥2.3 nmol/L, according to the upper limits of the accredited laboratory at Oulu University Hospital (NordLab) in fertile-aged women, and clinical hyperandrogenism (hirsutism) was diagnosed when a subject had a Ferriman–Gallwey (FG) score >7. Accordingly, women with polycystic ovaries (PCOs) in ultrasonography, oligo-amenorrhoea (OA) and hyperandrogenism (HA) (serum testosterone ≥2.3 nmol/L and/or FG score of >7) constituted 31.7% of the whole PCOS group, women with PCO and OA 60.4%, women with PCO and HA 4.7% and women with OA and HA 3.2%. Thus, 39.6% of women with PCOS were hyperandrogenic and 60.4% were normoandrogenic.

The control population consisted of women with normal appearing ovaries as assessed by ultrasonography and absence of PCOS-related symptoms (oligo- or amenorrhoea and/or hirsutism and/or elevated serum testosterone levels). Women using hormonal preparations and medications affecting glucose metabolism and steroid synthesis were excluded from the study. Alternatively, a washout period of 2 months was required for women using hormonal preparations before participating in the study. The diagnosis of pre-existing diabetes was an exclusion criterion in all studies, so none of the study subjects had type 2 diabetes. The samples were collected in a fasting state on any day of the menstrual cycle. A 2-h oral glucose tolerance test (OGTT) was carried out in 234 women with PCOS and in 109 non-PCOS controls. Fasting and two-hour glucose and insulin concentrations were measured after a 75 g glucose load. In the control group, 92.8% of the women had normal glucose tolerance (NGT) (fasting plasma glucose (FPG) ≤5.5 mmol/L or 2 h OGTT glucose <7.8 mmol/L) and 7.2% had impaired fasting glucose ((IFG); FPG 5.6–6.9 mmol/L) or impaired glucose tolerance ((IGT); 2 h OGTT glucose 7.8–11.0 mmol/L). In the PCOS group, 81.8% had NGT and 18.2% IFG/IGT. Informed consent was obtained from each subject after full explanation of the purpose and nature of all procedures used, and the study was approved by the Ethics Committee of Oulu University Hospital.

### Methods

Serum levels of RBP4 were analysed by enzyme-linked immunosorbent assay according to the manufacturer’s instructions (Quantikine ELISA; R&D Systems). The intra- and inter-assay coefficients of variation were 6.5 and 9% respectively. The metabolic variables (androstenedione (A), dehydroepiandrosterone sulphate (DHEAS), glucose, insulin, cholesterol, lipoproteins, triglycerides and high-sensitivity C-reactive protein) were analysed by means of routine methods used in the laboratories of the different study sites (22, 23, 24). Of note, the number of subjects varied between analyses owing to a lack of measurements in some cases. Serum concentrations of testosterone and sex hormone-binding globulin (SHBG) were analysed by means of liquid chromatography-mass spectrometry and chemiluminometric immunoassay, respectively, at Nordlab, Oulu, as reported earlier (26). The free androgen index (FAI) was calculated as testosterone/SHBG (both as nmol/L) × 100. Mean OGTT plasma glucose and serum insulin levels were calculated as the means of concentrations at different time points ((basal + 2-h)/2). Insulin resistance was defined by the homeostasis model assessment of insulin resistance (HOMA-IR) and insulin sensitivity by evaluating the composite insulin sensitivity index (ISI) or the Matsuda index as described earlier (27, 28).

### Statistical analysis

Statistical analyses were performed using SPSS 25.0 software (IBM Corp.). Variables with a skewed distribution were logarithmically transformed before statistical analysis. Differences between the PCOS and control groups were assessed using independent-samples *t*-tests. Adjustment for age and BMI was carried out by means of univariate general linear modelling using age and BMI as covariates. To evaluate the hormonal and metabolic changes with regard to age, the PCOS and control groups were grouped as follows: ≤30 years, 31–40 years and 41 years to menopause. One-way analysis of variance (ANOVA) with Tukey *post hoc* tests was used to assess the age-related changes of RBP4 between different age groups. Pearson’s correlation coefficients were used to assess the correlation between RBP4 and different variables, and adjustment for age and BMI was carried out by way of partial correlation analyses. Values of *P* < 0.05 were considered statistically significant.

## Results

### Characteristics of the study population

All anthropometric and metabolic parameters of the study population are shown in Table 1. Women with PCOS had a higher BMI compared with the controls. After adjusting for age and BMI, the levels of testosterone, FAI and A were significantly higher and those of SHBG lower in the PCOS group compared with the controls. Furthermore, women with PCOS had higher levels of triglycerides, fasting glucose, 2-h OGTT insulin and mean OGTT insulin and lower Matsuda indices compared with the non-PCOS controls after adjustment for age and BMI.

**Table 1:** Clinical, hormonal and metabolic parameters in control women and women with polycystic ovary syndrome.

Parameter	Control		PCOS		*P* value	*P* value adjusted*
	*n*	Mean (s.d.)	*n*	Mean (s.d.)		
Age (years)	191	33.0 (9.2)	278	32.4 (7.9)	0.511	–
BMI (kg/m^2^)	191	24.8 (4.9)	278	27.1 (5.7)	**<0.001**	–
WHR	191	0.80 (0.07)	278	0.82 (0.08)	**0.028**	0.901
Systolic BP (mmHg)	178	117 (13)	269	120 (15)	**0.027**	0.337
Diastolic BP (mmHg)	178	73 (10)	269	75 (11)	**0.040**	0.396
Testosterone (nmol/L)	191	0.9 (0.4)	278	1.4 (0.6)	**<0.001**	**<0.001**
SHBG (nmol/L)	191	56.2 (24.1)	278	46.6 (22.8)	**<0.001**	**0.008**
FAI	191	2.0 (1.4)	278	3.5 (2.2)	**<0.001**	**<0.001**
Androstenedione (nmol/L)	92	7.9 (4.4)	223	14.8 (8.4)	**<0.001**	**<0.001**
DHEAS (μmol/L)	58	3.9 (1.8)	223	4.8 (2.7)	0.050	0.188
Total cholesterol (mmol/L)	176	4.5 (0.9)	106	4.8 (1.0)	**0.013**	0.803
HDL (mmol/L)	176	1.6 (0.3)	106	1.5 (0.4)	0.549	0.784
LDL (mmol/L)	176	2.5 (0.9)	106	2.8 (0.9)	**0.001**	0.920
Triglycerides (mmol/L)	176	0.9 (0.4)	106	1.1 (0.7)	**<0.001**	**0.024**
hs-CRP (mg/L)	138	1.3 (2.5)	244	2.3 (3.3)	**<0.001**	0.974
Fasting glucose (mmol/L)	167	4.8 (0.5)	275	5.0 (0.5)	**<0.001**	**0.002**
Fasting insulin (mIU/L)	167	7.4 (5.4)	275	9.2 (6.8)	**0.003**	0.798
OGTT glucose, 2 h (mmol/L)	109	4.9 (1.2)	234	5.6 (1.5)	**<0.001**	0.104
OGTT insulin, 2 h (mIU/L)	109	29.0 (22.7)	234	62.2 (60.9)	**<0.001**	**0.020**
OGTT mean glucose (mmol/L)	109	4.9 (0.7)	234	5.3 (0.9)	**<0.001**	0.086
OGTT mean insulin (mIU/L)	109	18.2 (13.2)	234	35.9 (33.1)	**<0.001**	**0.045**
HOMA-IR	167	1.6 (1.2)	275	2.1 (1.7)	**<0.001**	0.507
Matsuda index	109	12.0 (8.3)	234	7.9 (6.5)	**<0.001**	**0.039**
RBP4 (mg/L)	191	33.5 (18.3)	278	45.1 (24.0)	**<0.001**	**<0.001**

Data shown as mean (s.d.). Statistically significant *P* values are in bold.

*P values adjusted for age and BMI using univariate general linear modelling.

BMI, body mass index; BP, blood pressure; DHEAS, dehydroepiandrosterone sulphate; FAI, free androgen index; HDL, high-density lipoprotein; HOMA-IR, homeostatic model assessment of insulin resistance; hs-CRP, high-sensitivity C-reactive protein; LDL, low-density lipoprotein; OGTT, oral glucose tolerance test; PCOS, polycystic ovary syndrome; RBP4, retinol-binding protein 4; SHBG, sex hormone-binding globulin; WHR, waist-hip ratio.

Clinical, hormonal and metabolic variables in the different age groups are shown in Table 2. The levels of testosterone, FAI, A, DHEAS and triglycerides were significantly higher and those of SHBG lower in women with PCOS aged ≤30 years compared with controls in the same age group after adjusting for BMI. In the age group of 31–40 years, the levels of testosterone, FAI, A, fasting glucose, 2-h OGTT insulin and mean OGTT glucose were higher in the PCOS group after adjusting for BMI. Furthermore, the waist-hip ratio (WHR) and the FAI were significantly higher in women with PCOS in the age group of 41 years to menopause.

**Table 2:** Clinical, hormonal and metabolic parameters in control women and women with polycystic ovary syndrome in different age groups.

Parameter	≤30 years			31–40 years			41–menopause		
	Control	PCOS	*P* value*	Control	PCOS	*P* value*	Control	PCOS	*P* value*
Age	24.5 (2.9)	26.2 (2.7)	<0.001	36.5 (2.8)	35.4 (2.7)	0.031	45.4 (3.3)	44.6 (3.3)	NS
BMI (kg/m^2^)	23.0 (4.2)	26.1 (5.7)	–	26.8 (5.5)	27.9 (5.6)	–	25.8 (4.3)	28.4 (5.4)	–
WHR	0.78 (0.05)	0.80 (0.07)	NS	0.83 (0.09)	0.82 (0.07)	NS	0.81 (0.06)	0.87 (0.09)	0.021
Systolic BP (mmHg)	112 (10)	115 (12)	NS	121 (14)	123 (14)	NS	124 (15)	130 (19)	NS
Diastolic BP (mmHg)	68 (7)	71 (9)	NS	76 (11)	78 (10)	NS	78 (10)	82 (11)	NS
Testosterone (nmol/L)	0.9 (0.4)	1.5 (0.6)	<0.001	1.0 (0.4)	1.4 (0.8)	<0.001	1.0 (0.5)	1.0 (0.4)	NS
SHBG (nmol/L)	61.5 (23.9)	48.7 (23.5)	0.043	48.9 (21.7)	45.2 (22.1)	NS	55.2 (25.1)	42.9 (21.6)	NS
FAI	1.8 (1.4)	3.7 (2.2)	<0.001	2.4 (1.6)	3.7 (2.6)	0.002	2.0 (0.9)	2.8 (1.7)	0.034
Androstenedione (nmol/L)	8.5 (4.6)	15.9 (8.7)	<0.001	4.2 (1.8)	14.1 (8.5)	0.001	7.4 (3.0)	9.8 (3.7)	NS
DHEAS (μmol/L)	3.5 (1.5)	5.2 (2.9)	0.008	5.0 (2.2)	4.2 (2.0)	NS	3.3 (1.3)	4.0 (2.2)	NS
Total cholesterol (mmol/L)	4.3 (0.9)	4.2 (0.7)	NS	4.7 (0.8)	4.7 (0.9)	NS	4.9 (1.0)	5.2 (1.0)	NS
HDL (mmol/L)	1.5 (0.3)	1.4 (0.3)	NS	1.6 (0.3)	1.4 (0.3)	NS	1.7 (0.4)	1.7 (0.5)	NS
LDL (mmol/L)	2.2 (0.9)	2.4 (0.6)	NS	2.7 (0.7)	2.8 (0.9)	NS	2.9 (1.0)	3.1 (0.9)	NS
Triglycerides (mmol/L)	0.7 (0.3)	1.0 (0.5)	0.007	1.0 (0.4)	1.1 (0.7)	NS	1.0 (0.5)	1.2 (0.7)	NS
hs-CRP (mg/L)	1.2 (3.0)	2.5 (3.6)	NS	1.5 (1.8)	2.2 (3.3)	NS	1.4 (1.6)	1.8 (2.4)	NS
Fasting glucose (mmol/L)	4.9 (0.5)	5.0 (0.5)	NS	4.7 (0.5)	5.1 (0.6)	0.001	4.9 (0.5)	5.1 (0.7)	NS
Fasting insulin (mIU/L)	7.1 (4.5)	9.0 (6.6)	NS	8.6 (6.7)	9.8 (8.1)	NS	6.8 (5.5)	9.0 (5.5)	NS
OGTT glucose, 2 h (mmol/L)	4.8 (1.0)	5.5 (1.4)	NS	4.7 (1.1)	5.8 (1.7)	NS	5.1 (1.5)	5.4 (1.5)	NS
OGTT insulin, 2 h (mIU/L)	34.5 (19.6)	61.8 (55.9)	NS	22.0 (13.9)	66.9 (70.1)	0.040	26.8 (30.1)	57.7 (62.2)	NS
OGTT mean glucose (mmol/L)	5.0 (0.6)	5.3 (0.8)	NS	4.7 (0.6)	5.5 (1.0)	0.014	5.0 (0.9)	5.2 (0.9)	NS
OGTT mean insulin (mIU/L)	21.1 (11.1)	35.4 (30.6)	NS	14.9 (9.4)	38.9 (38.3)	NS	16.9 (17.7)	33.6 (33.2)	NS
HOMA-IR	1.5 (0.9)	2.1 (1.6)	NS	1.8 (1.5)	2.3 (2.1)	NS	1.5 (1.2)	2.0 (1.2)	NS
Matsuda	9.0 (3.9)	7.6 (5.3)	NS	13.6 (8.3)	7.8 (7.1)	NS	15.1 (11.1)	8.5 (8.3)	0.046
RBP4 (mg/L)	27.1 (10.4)	47.7 (23.5)	<0.001	38.1 (21.3)	42.1 (22.5)	NS	40.5 (22.5)	42.3 (26.6)	NS

Data shown as mean (s.d.).

*P values adjusted for BMI in individual age groups using univariate general linear modelling.

BMI, body mass index; BP, blood pressure; DHEAS, dehydroepiandrosterone sulphate; FAI, free androgen index; HDL, high-density lipoprotein; HOMA-IR, homeostatic model assessment of insulin resistance; hs-CRP, high-sensitivity C-reactive protein; LDL, low-density lipoprotein; OGTT, oral glucose tolerance test; PCOS, polycystic ovary syndrome; RBP4, retinol-binding protein 4; SHBG, sex hormone-binding globulin; WHR, waist-hip ratio.

### Serum levels of RBP4 based on age and BMI stratification

**Figure 1:**
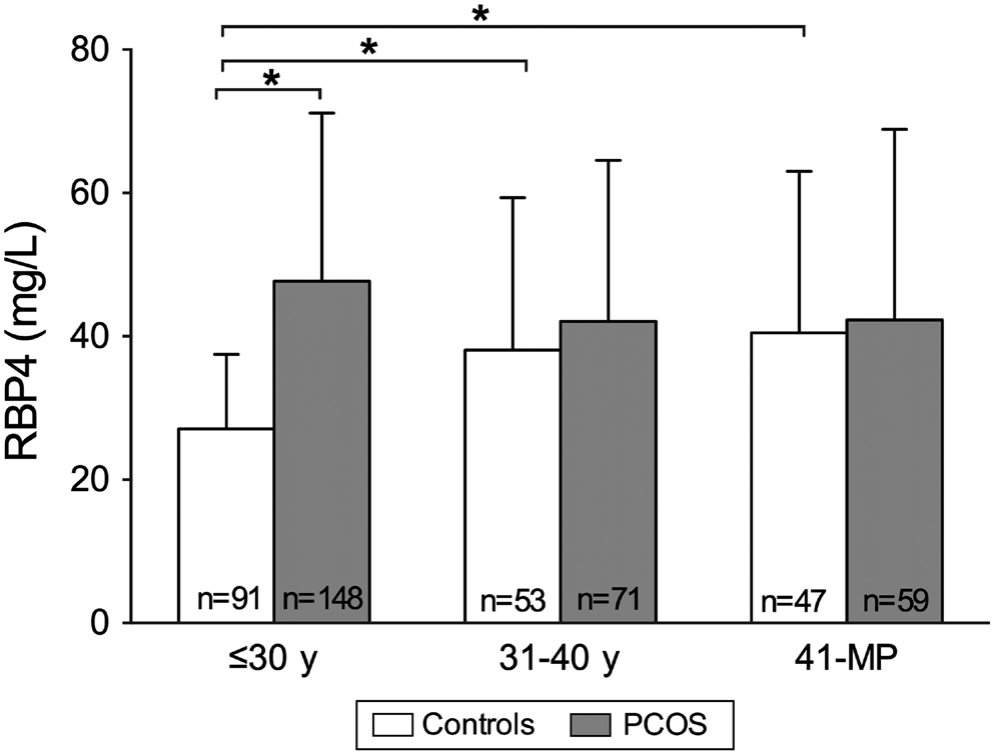
Concentrations of serum RBP4 in control women and in women with polycystic ovary syndrome in different age groups. The *bars* represent means and the *error bars* standard deviation. *n* denotes the number of subjects. *P < 0.001.

**Figure 2:**
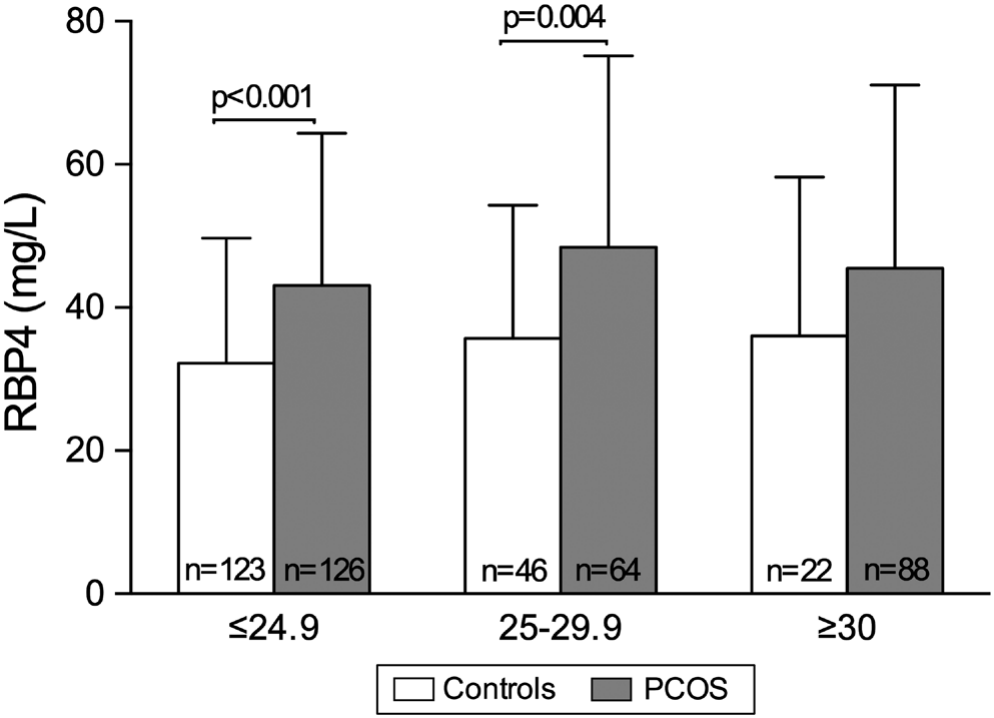
Concentrations of serum RBP4 in control women and in women with polycystic ovary syndrome in different BMI groups. The *bars* represent means and the *error bars* standard deviation. BMI in kg/m^2^. *n* denotes the number of subjects.

In the whole study population, the concentrations of RBP4 were increased in the PCOS group compared with controls after adjusting for age and BMI. Age-stratified analysis showed that this increase in serum RBP4 levels was observed only in women with PCOS aged ≤30 years compared with controls, but not in the other age groups (Table 2), and this was also the case after adjustment for BMI. One-way ANOVA indicated that serum concentrations of RBP4 increased with age up to menopause in the control group (*P* < 0.001), whereas the levels remained unchanged in women with PCOS (*P* = 0.106) (Fig. 1). When the subjects were divided into different BMI groups (normal weight <25 kg/m^2^, overweight 25–30 kg/m^2^ and obese >30 kg/m^2^), the concentrations of RBP4 were increased in both lean and overweight women with PCOS after adjusting for age (Fig. 2). Furthermore, a subanalysis was performed after excluding women with PCOS aged over 46 years as these women exhibited lowered androgen levels. The results were similar to those found in the whole study population (data not shown).

### Serum levels of RBP4 based on phenotype, androgen status and glucose tolerance

**Figure 3:**
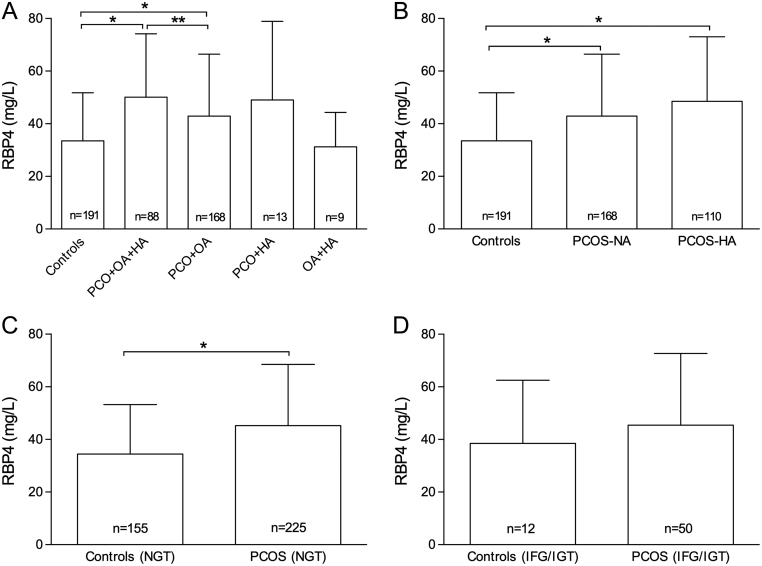
Concentrations of serum RBP4 in control women and in women with polycystic ovary syndrome. The *bars* represent means and the *error bars* standard deviation. n denotes the number of subjects. *P < 0.001, **P < 0.05. (A) women with PCOS with different phenotypes; (B) normoandrogenic (NA) and hyperandrogenic (HA) women with PCOS; (C) normal glucose tolerant (NGT) subjects; (D) impaired fasting glucose (IFG)/impaired glucose tolerant (IGT) subjects.

Serum RBP4 levels were increased in women with PCOS in the PCO + OA + HA and PCO + OA phenotype groups when compared with the control women (Fig. 3A). Furthermore, women with PCO + OA + HA had increased levels of serum RBP4 levels when compared with those with PCO + OA. These results remained significant even after adjustment for age and BMI. The RBP4 levels in the other two phenotypes (PCO + HA, OA + HA) could not be statistically compared with others as the numbers of subjects in these groups were few.

Serum levels of RBP4 were increased in both normoandrogenic and hyperandrogenic women with PCOS when compared with the control women after adjusting for age and BMI (Fig. 3B). Women with PCOS and NGT had higher levels of serum RBP4 when compared with controls with NGT (Fig. 3C), whereas no statistically significant differences were observed in the IFG/IGT subjects (Fig. 3D). Further, no differences were found in the serum levels of RBP4 in the NGT vs IFG/IGT controls, which was also the case in women with PCOS (data not shown).

### Correlation analyses

Serum concentrations of RBP4 were weakly negatively correlated with levels of serum fasting glucose in the PCOS group after adjustment for age and BMI (*r* = −0.229, *P* < 0.001). No significant correlations with either steroids or lipids were observed in the whole PCOS group. In the control group, levels of RBP4 were positively correlated with age (*r* = 0.376, *P* < 0.001). No other statistically significant correlations were seen in the control group after age and BMI adjustment.

In women with PCOS aged ≤30 years, levels of RBP4 were positively correlated with WHR (*r* = 0.193, *P* = 0.020) and triglyceride levels (*r* = 0.685, *P* = 0.001) and negatively correlated with fasting glucose levels (*r* = −0.252, *P* = 0.002) after BMI adjustment.

## Discussion

The present study showed that serum RBP4 levels were higher in young women with PCOS (≤30 years of age) when compared with their age-matched non-PCOS controls. Furthermore, RBP4 levels remained unchanged with age in the PCOS group while in control women they increased up to menopause.

Conflicting results have been reported as regards levels of RBP4 and its role in the pathogenesis of PCOS and insulin resistance. In line with the present results, higher levels of RBP4 in women with PCOS have been reported in some (12, 29, 30) but not all studies (15, 16, 31).

Earlier studies have been carried out to investigate the association between RBP4 levels and various anthropometric indices including BMI and WHR. Consistent with our results, RBP4 levels have previously been shown to correlate positively with WHR, but not with BMI (12). In contrast, two studies revealed no correlation between RBP4 and WHR (13, 15). In addition, one study revealed that RBP4 levels were positively correlated with age in controls, but not in women with PCOS (32). Furthermore, consistent with the results of an earlier study (32), we found increased levels of RBP4 in lean women with PCOS compared with controls with similar BMIs. In contrast, in another study no difference was found in the levels of RBP4 between lean women with and without PCOS, but higher levels in obese women with PCOS vs their controls (18).

Previous studies have shown that RBP4 is associated with fatty acid metabolism and there is a strong association between RBP4 and hypertriglyceridaemia (15, 18, 29). However, some other studies have not revealed such an association (30, 32, 33). In the present study, triglyceride levels were positively correlated with those of RBP4 in younger women with PCOS, suggesting that elevated RBP4 levels might arise from altered triglyceride metabolism. Furthermore, larger adipocyte size in women with PCOS compared with non-PCOS women (34) may also play a role in increased RPB4 levels.

Elevated androgen levels are a key feature in women with PCOS, and the association of increased RBP4 levels with androgen levels could have implied that androgens may contribute to the increased serum levels of RBP4 observed. However, this was not the case as we found no correlations between RBP4 and any of the measured androgens in the present study. This is in line with the results of other studies, which have revealed no association between elevated RBP4 levels and androgens (12, 17). Conversely, another study showed positive correlations between circulating concentrations of testosterone, DHEAS, A and RBP4 (35). In the present study, women with PCO + OA + HA had higher levels of serum RBP4 when compared with those with PCO + OA. According to previous literature, phenotypes with HA are considered metabolically more severe compared with those without HA (36). However, it has to be noted that we could not compare serum RBP4 levels in women with other phenotypes (PCO + HA and OA + HA), as the numbers of such women were few.

A few previous studies have demonstrated that RBP4 levels are strongly correlated with HOMA-IR in women with PCOS (11, 34). One study revealed that RBP4 levels correlated less strongly with insulin resistance in women with PCOS, although PCOS cases and controls showed no differences in RBP4 levels (16). These results are in contrast with those reported in other studies, which did not show any significant association between elevated RBP4 levels and insulin or insulin resistance as measured by HOMA (15, 35). Likewise, our results showed that increased RBP4 levels do not correlate with insulin resistance as measured by HOMA-IR, suggesting that increased RBP4 levels observed in this study are not attributable to insulin resistance *per se* and serum concentrations of RBP4 might not directly affect glucose metabolism.

The heterogeneity of results obtained in studies of RBP4 in women with PCOS may be attributed to factors including different cohorts studied (obese vs non-obese; normoandrogenic vs hyperandrogenic; normal vs IGT; population-based vs hospital-based study population), differences in criteria in selection of women with PCOS (either Rotterdam or NIH criteria), methodological differences in measurements of RBP4 levels (Western blot vs ELISA) and differences in the methodologies used for assessing insulin resistance (OGTTs vs clamp studies) (15). In addition, there are different polymorphisms of RBP4, which may influence the association between RBP4 and insulin resistance (37).

There are several strengths in the present study. Our study included a well-characterised relatively large PCOS cohort ranging from a young age up to menopause, which enabled detailed evaluation of changes in hormonal and metabolic parameters, and their association with serum RBP4 levels. Even though the study subjects were recruited at different sites, all diagnoses of PCOS were made according to Rotterdam criteria. We addressed the issue of heterogeneity of results obtained in studies of RBP4 in women with PCOS by comparing serum levels of RBP4 in women with different phenotypes of PCOS, according to their androgen status and also according to their glucose tolerance. A limitation of the study is that we were unable to assess the effect of menstrual cycle changes on serum RBP4 levels, as the phase of the menstrual cycle in 25% of the controls and 45% of the women with PCOS could not be ascertained, while the rest of the samples were taken in the follicular phase. However, the results remained the same when the samples taken in the follicular phase were analysed separately. Furthermore, the washout period for hormonal contraceptives was 2 months, which might have influenced the levels of RBP4, although an earlier study has shown that RBP4 levels are not influenced by oral contraceptive pills (15).

In conclusion, even though RBP4 has been shown to reflect disturbances in glucose metabolism in previous studies in the general population, and we found higher serum levels in younger women with PCOS, we were not able to establish a role of RBP4 in detecting metabolic derangements in PCOS in clinical practice.

## Declaration of interest

The authors declare that there is no conflict of interest that could be perceived as prejudicing the impartiality of the research reported.

## Funding

This work was supported by grants from the Sigrid Jusélius Foundation, Päivikki and Sakari Sohlberg Foundation, Medical Research Centre Oulu, Oulu University Hospital and the University of Oulu.
